# Selective impairment of emotion recognition through music in Parkinson's disease: does it suggest the existence of different networks for music and speech prosody processing?

**DOI:** 10.3389/fnins.2013.00161

**Published:** 2013-09-12

**Authors:** Tobias A. Mattei, Abraham H. Rodriguez, Juri Bassuner

**Affiliations:** ^1^Neurosurgery Department, Ohio State UniversityColumbus, OH, USA; ^2^Department of Surgery/Division of Neurosurgery, University of MissouriColumbia, MO, USA; ^3^Department of General Medicine, School of Medicine, University of MissouriColumbia, MO, USA

**Keywords:** music therapy, emotion recognition, Parkinson disease, speech prosody, non-motor symptoms, rehabilitation

Although the main hallmark of Parkinson's disease (PD) are its motor symptoms, such as bradykinesia, resting tremor, postural instability, and rigidity, it has been increasingly recognized that such disease constitutes, in fact, a complex degenerative process which presents a variety of different clinical manifestations. Actually, in the last years, significant attention has been devoted to the so-called non-motor symptoms of PD, especially psychiatric ones, such as anxiety, depression, apathy, dysphoria and irritability, which have been shown to be present even in the early stages of disease (Leroi et al., 2012) and which show increasing intensity with its progression. Similarly, sleep disorders (such as difficulty for initiating sleep, frequent night awakenings, nocturia, restless legs syndrome, apnea, parasomnias and increased daytime sleepiness) have already been shown to occur with increased prevalence in patients with PD in comparison to the healthy population (Raggi et al., 2013). Nevertheless, the literature on the effects of PD upon other higher cognitive features, such as musical processing, is still scarce.

Motivated by such increased attention to non-motor symptoms PD, and with basis on previous physiological studies which have demonstrated that the perception of music rhythm and beat is mediated mainly by the basal ganglia (the major anatomical complex involved in the etiology of PD) (Grahn, 2009), several recent studies have investigated the effects of PD over music perception. For example, it has already been shown that patients with PD have reduced capacity of synchronizing movements to a beat and, in turn, discriminating changes in tempo (“faster” vs. “slower”) (Grahn and Brett, 2009). Interestingly, such difficulty has been shown to be more marked when the beat is introduced at a slower tempo and progressively speeded up (McAuley et al., 2012). In such study, however, the capacity of patients with PD of discriminating changes in non “beat-based” rhythms did not significantly differ from that of a healthy control group. Ultimately, by correlating the impairment in beat perception with a decline in the coordinated functional activity between the basal ganglia, thalamus, premotor and supplementary motor regions, such studies have underscored the importance of the motor circuitry in beat perception.

Nevertheless, in a recently published study, Lima et al. (2013) demonstrated that the effects of PD upon music perception may not be limited to rhythm detection, but may also involve the recognition of emotions as expressed through music. The authors of such investigation have demonstrated that patients with PD had increased difficulty to recognize happiness and peacefulness, when combinedly expressed through music lyrics and rhythm, while presenting intact perception of sadness and fear. Comparatively, in relation to non-musical speech, such patients presented only a mild global impairment of emotion recognition which seemed to be more a result of an executive dysfunction that a direct effect of the disease over the limbic system. Similarly, other previous investigations had already demonstrated that a dysfunction in the dopaminergic system may only partially explain the observed impairment in emotion recognition through facial expressions in patients with PD (Bediou et al., 2012), as the treatment with levodopa did not significantly modify such deficit. This finding was further confirmed by a recent meta-analysis on the issue (Gray and Tickle-Degnen, 2010). Conversely, in studies evaluating the effects of surgical treatment for Parkinson disease with deep brain stimulation (DBS), a therapeutic strategy which has been suggested to have broader effects in terms of modulating several interconnected circuits and not only the deep basal ganglia, it was possible to observe a synergistic improvement in the ability of recognizing the emotional content of facial expressions after combined treatment with both DBS and levodopa (Mondillon et al., 2012).

In another study which also demonstrated impaired recognition of emotions as expressed through music in patients with PD, it was possible to observe that, although the observed deficit in fear recognition was at least partially associated with some degree of executive dysfunction, the deficit in emotion recognition in patients with PD persisted even after adjusting for executive functioning levels (van Tricht et al., 2010). Additionally, despite the fact that a previous study has suggested that depressed patients may have a negative emotional bias when evaluating musical stimuli (Punkanen et al., 2011), in this study the observed deficit in emotion recognition through music was not related to depressive symptoms, disease duration or severity of motor symptoms. All these results suggest that, although the observed impairment of emotion recognition through music in patients with PD may be partially related to a certain degree of executive dysfunction, it most likely reflect a separate primary cognitive deficit in emotional processing.

The suggestion that it may be possible to localize specific cognitive features of emotional and music processing to single anatomical areas of the brain has been investigated by some recent studies which evaluated the neuroanatomical regions activated by facial and music recognition in patients with semantic dementia and Alzheimer's disease (Omar et al., 2011). The results of such investigations demonstrated that the neurocognitive process involved in emotion recognition through facial expressions and music seems to be mediated by different but interconnected networks, with specific patterns of activation depending on the familiarity of the presented sensorial stimuli. In practical terms, the anterior tip of the right anterior temporal lobe seems to directly correlated with the ability to recognize famous tunes and famous faces, while the ability of identifying everyday tunes seems to activate the right mesial temporal structures, especially the amygdala (Hsieh et al., 2011).

In opposition to the presented evidence which support the thesis that music and speech prosody may be mediated by different neurophysiological networks, several previous investigations have demonstrated a very close relationship between music perception and prosody (Thompson et al., 2004; Hutchins et al., 2010; Escoffier et al., 2013). Therefore, the verification of isolated effects of PD upon emotional processing through music perception (but not prosody) may consist in either a bias of the employed methodological approach, or an isolated effect related to motor-related rhythm dysfunction in patients with PD, rather than a true evidence of the existence of distinct networks for processing emotions (one musical and one non-musical) in normal physiological situations. Ultimately, further clinical and imaging studies are still required in order to confirm the validity of generalizing such findings in patients with PD to the healthy population.

Finally, from a methodological standpoint, the findings of Lima et al. study suggest that future attempts of investigating the effects of degenerative diseases (as well as their treatment) upon the cognitive psychology of emotion processing should focus on specific neuropsychological domains (Hsieh et al., 2012), as there might be significant differences among the effects of such diseases upon recognition of emotional content from specific sensory inputs (for example, through visual or auditory modalities), and even among specific variations within one specific sensory modality (such as the difference between emotional recognition through musical and non-musical speech).
